# Incidence of myeloproliferative neoplasms in Calgary, Alberta, Canada

**DOI:** 10.1186/s13104-019-4321-1

**Published:** 2019-05-24

**Authors:** Jonathan Heppner, Leonard Tu Nguyen, Maggie Guo, Christopher Naugler, Fariborz Rashid-Kolvear

**Affiliations:** 1 College of Medicine, University of Saskatchewan, Saskatoon, SK Canada; 2 Department of Pathology and Laboratory Medicine, Cumming School of Medicine, University of Calgary, Calgary, AB Canada; 3 Alberta Public Laboratories (formerly Calgary Laboratory Services), Calgary, AB Canada; 4 Departments of Family Medicine and Community Health, Cumming School of Medicine, University of Calgary, Calgary, AB Canada; 5 Department of Medical Genetics, Cumming School of Medicine, University of Calgary, Calgary, AB Canada

**Keywords:** Myeloproliferative neoplasm, Essential thrombocythemia, Polycythemia vera, Primary myelofibrosis, Cancer epidemiology, Hematological neoplasm

## Abstract

**Objective:**

The incidence of the combined myeloproliferative neoplasms (MPNs) was determined for a major Canadian city. Retrospective cases of MPN diagnoses (essential thrombocythemia, polycythemia vera, and primary myelofibrosis) between 2011 to 2015 were retrieved from the Southern Alberta Cancer Cytogenetics Laboratory’s database at Alberta Public Laboratories.

**Results:**

An incidence rate of 2.05 cases per 100,000 person-years (95% CI 1.73–2.41) was determined, giving an age-standardized Canadian incidence of 2.71 cases per 100,000 person years (95% CI 2.63–2.78). MPN diagnoses occurred at a wide age range of 8–93 (median 66) and an age-dependent increase in incidence. Incidence rates for the MPNs are first reported here for a Canadian population.

## Introduction

The myeloproliferative neoplasms (MPNs) are clonal myeloid malignancies of the bone marrow classified by their unique genetic etiology and hematological histomorphologic features. The broadest classification is by BCR-ABL (Philadelphia chromosome) positive status for chronic myeloid leukemia (CML). The BCR-ABL negative MPNs, frequently characterized by mutations in Janus kinase 2 (JAK2), are further subclassified into essential thrombocythemia (ET), polycythemia vera (PV), primary myelofibrosis (PMF), and unclassifiable MPN [1]. Recently, mastocytosis was removed from classification as an MPN and while it has clinical and pathological features of an MPN, chronic myelomonocytic leukemia has been reclassified as a myelodysplastic syndrome/MPN disorder. Establishing clear discerning criteria between MPN classes requires summation of findings from peripheral blood slides, bone marrow morphology, cytogenetic and genetic alterations, and complete blood count [1, 2]. Specific classification is essential not only for appropriate intervention strategies, but also prognostication. While myelodysplastic syndrome and acute myelogenous leukemia are not classified as MPNs, they are common transformations of the MPNs associated with significantly increased mortality, illustrating the need for appropriate diagnosis and prognosis.

Although the MPNs are rare cancers relative to solid tumors, they are an increasingly prevalent global healthcare issue and their epidemiology have been reported in the United States, Europe, and the Republic of Korea [3–7]. ET and PV consistently make up the majority of MPN cases in all populations studied with the mean age of diagnosis in the 50 to 60 range [4–7]. However, it has been noted that assessment of global incidence has been hampered by inconsistencies in diagnostic capacities and reporting between countries [3]. Furthermore, as diagnostic criteria change with increasing understanding of pathogenesis, so too will incidence and prevalence estimates. For example, it has been suggested that discovery of the JAK2 genetic mutation, along with improved testing diagnostic capacity, are factors potentially explaining increased prevalence and incidence over time [4]. There is also evidence of increased diagnostic stringency potentially decreasing perceived incidence [7], presumably through reduction of false positive results. Furthermore, MPN incidence is expected to rise in aging populations. Accordingly, monitoring the epidemiology of MPNs is essential to follow trends in population health, allocate appropriate healthcare resources, and evaluate the impact of ongoing diagnostic criteria refinement.

To date, there have been no reports on the incidence of the MPNs in the Canadian population. As a complement to the reported incidence of CML [8], we present the collective incidence of BCR-ABL-negative MPNs in Calgary, Alberta as well as their subclasses.

## Main text

### Methods

#### Ethics approval

This study was approved by the Health Research Ethics Board of the Alberta Cancer Committee (ID HREBA.CC-16-0830).

#### Data source

Patient data for this retrospective study was obtained from the Cancer Cytogenetics Laboratory at Alberta Public Laboratories (formerly Calgary Laboratory Services), which is the clinical testing facility serving the catchment area of over 1.8 million residents in Calgary and the surrounding Southern Alberta region. Blood and bone marrow were classified according to the 2008 WHO guidelines via flow cytometry and hematopathologic examination [9]. Incident MPN cases were identified for the period of January 1, 2011 until December 31, 2015, and pathology reports were analyzed to determine MPN subclass as either ET, PV, PMF or unclassified MPN. ET is distinguished by sustained thrombocytosis (platelet count ≥ 450 × 10^9^/L), and proliferation and maturation of megakaryocytes with no known genetic or biological marker. PV is distinguished by elevated RBC production (measured by haemoglobin concentration, haematocrit, or RBC mass) with bone marrow biopsy showing hypercellularity with trilineage growth (erythroid, granulocytic, and megakaryocytic) and the *JAK2* V617F mutation. PMF is distinguished by the proliferation of atypical megakaryocytes and granulocytes which may be accompanied by reticulin or collagen fibrosis in later development.

#### Case identification

Crude incidence rates were calculated for MPN cases in sex and 5-year age categories, and the age-standardized incidence for Canada was calculated using published methods [10]. Population estimates determined by post-censal coverage studies were retrieved from the Canadian Socio-Economic Information Management System (CANSIM) database from Statistics Canada [11, 12]. 95% confidence intervals were calculated for each incidence rate by Wilson score interval for binomial proportions [13] using cumulative 2011–2015 population totals for Calgary and Canada, respectively.

### Results

From 2011 to 2015, 139 new MPN diagnoses were made in the Calgary Metropolitan area, giving a crude incidence of 2.05 cases per 100,000 person-years (95% CI 1.73–2.41, Table 1). There was slight gender bias with a male to female ratio of 1.14. The range for age at diagnosis was broad from 8 to 93 with a median of 66. Between the subclasses, ET had the highest incidence followed by PMF, PV, and unclassified MPN (MPN-U). The age-standardized incidence for Canada was 2.71 cases per 100,000 person-years (95% CI 2.63–2.78).

**Table 1:** Incidence rates and features of myeloproliferative neoplasms in Calgary (2011–2015)

	ET	PV	PMF	MPN-U	All MPN
New cases per year	12.4	4.4	7.6	3.4	27.8
M/F	0.94	0.93	3.75	0.31	1.14
Median age of diagnosis	61	67	71	57	66
Age range	8–93	13–88	37–92	14–92	8–93
Crude incidence per 100,000 person-years	0.92	0.32	0.56	0.25	2.05
(95% CI)	(0.71–1.16)	(0.21–0.48)	(0.40–0.76)	(0.15–0.39)	(1.73–2.41)
Age-standardized incidence for Canada per 100,000 person-years	1.16	0.44	0.80	0.31	2.71
(95% CI)	(1.11–1.21)	(0.41–0.47)	(0.76–0.84)	(0.28–0.33)	(2.63–2.78)

ET, essential thrombocythemia; PV, polycythemia vera; PMF, primary myelofibrosis; MPN-U: myeloproliferative neoplasm-unknown; MPN, myeloproliferative neoplasm

**Fig. 1:**
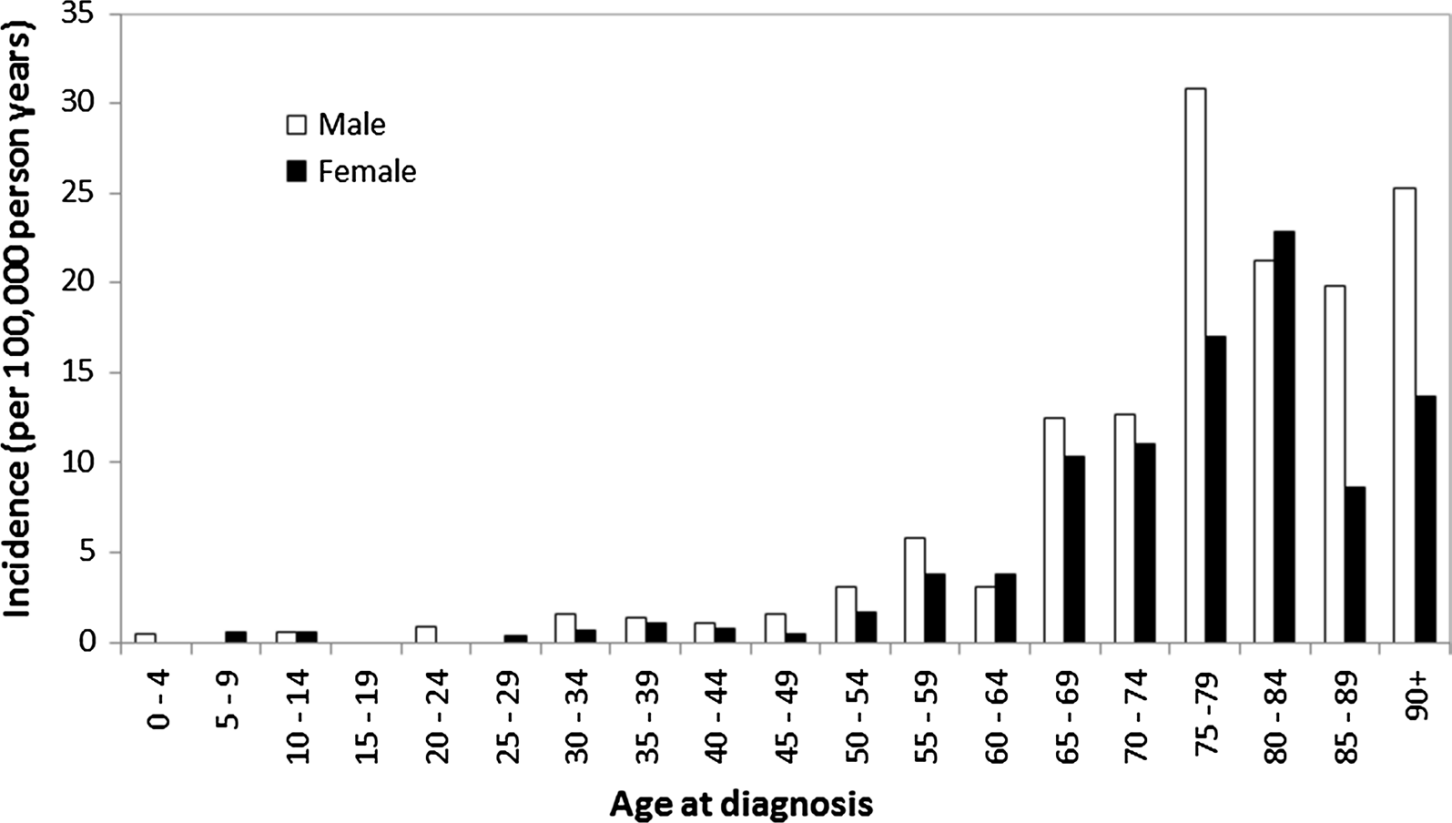
Age and sex-categorized incidence rates of myeloproliferative neoplasms in the Calgary metropolitan area (2011–2015)

The distribution of age- and sex-categorized MPN incidence rates is shown in Fig. 1. New cases were observed at early age in a few children, however there is a dramatic rise for patients older than 50. The highest incidence was observed later in life past 75 years, particularly for males.

### Discussion

The crude incidence reported here for myeloproliferative neoplasms in Calgary and standardized rate for Canada, which has a proportionally older population, are generally lower than corresponding values from other regions including the United States (Table 2). A direct comparison may not be straightforward due the changing diagnostic criteria of MPNs and population-specific factors such as age and genetic variance. Furthermore, incidence rates may be variable both between close populations as well as within populations over time [4, 6, 7]. Changing populations and refinements in diagnostic sensitivity, criteria, and reporting are thought to contribute to the increasing MPN incidence rate over the past decades.

**Table 2:** Incidence rates (per 100,000 person-years) of MPNs

Region	Period	Subclass				MPN	Source
		ET	PV	PMF	MPN-U		
Canada	2011–2015	1.16	0.44	0.80	0.31	2.71 (non-CML), 3.58 (all)	Current study, [8]
USA	2008–2011	0.9	1.0	0.3	NA	4.2 (all)	SEER [4]
Norway	2010–2012	1.0	0.7	0.5	0.1	2.9 (non-CML)	[5]
Europe (18 countries)	1995–2002	0.4	0.5	0.1	NA	NA	[6]
Korea	2004–2013	2.4	1.2	0.4	NA	NA	[7]

ET, essential thrombocythemia; PV, polycythemia vera; PMF, primary myelofibrosis; MPN-U, myeloproliferative neoplasm-unknown; NA, not available; SEER, surveillance, epidemiology, and end results registry

The slight bias of MPNs for males over females is in agreement with the literature [3], however there is some variance between subtypes. There was almost four times as many male cases of PMF than female and the gender preference is flipped for the other subclasses. In literature values, the high proportion of male PMF incidence was also observed but not as strongly as observed here. Female preference in ET cases has also been remarked, however male incidence has been generally found to be more dominant for PV [4, 6, 7]. With a median age of diagnosis of 71, PMF incidence was generally older than the other subclasses. Age bias in the MPNs has been discussed; for example a recent report of ET had the highest incidence compared to other MPNs in a USA population under 40 years of age [14]. The age of diagnosis for ET cases in Calgary reported here has a relatively low median of 61, however the highest incidence still occurs in the advanced age group of 80–84 years. In light of the sex and age biases MPN incidence, it would be interesting to examine the relationship between age of diagnosis and sex ratio in future studies.

The calculated Canadian incidence rates for the MPN subtypes were near the range of published values for other regions (Table 2) except for PMF which was markedly high at 0.80 cases per 100,000 person-years. The corresponding crude incidence was 0.56 cases per 100,000 person-years. The elevated age-standardized rate may be largely attributed to Calgary having a relatively younger population and diagnoses occurring at advanced age, with 29 of the 38 PMF cases in adults 65 years or older. Relative differences in incidence of the MPNs between countries are partially due to inherent genetic and environmental factors, however variation in healthcare systems, diagnostic practice and reporting should also be taken in mind.

Unclassifiable MPN is defined as a heterogenous mix of incomplete diagnoses that cannot be differentiated into one of the classic subtypes, usually because the disease is in early stage. Here, MPN-U accounted for 17 cases of 139 MPN (12.2%), which falls within the estimated range of 10–15% [15]. The proportion of MPN-U’s is expected to decrease as differential diagnostic criteria are undergoing refinement, especially in light of the updated 2017 WHO diagnostic criteria which are primarily focused on differentiating the MPN subtypes [16]. In a retroactive reclassification study using the 2016 criteria, the size of the MPN-U category was reduced by about 30% [17].

In the 5 years study period, three cases of MPN were observed in patients under 20 years old, the youngest at 8 years old. Early-onset disease is typically suggestive of genetic predisposition, however familial cases of MPN are very rare and the epidemiology of early-onset MPN is mostly unknown [18].

In summary we report for the first time a cumulative MPN incidence of 2.05 cases per 100,000 person-years in Calgary and an age-standardized rate of 2.71 cases per 100,000 person-years for Canada. This is in general agreement with literature values, however a high rate of primary myelofibrosis was observed, especially in males. Future studies should be conducted to further refine these estimates and build upon the epidemiology of the MPNs.

## Limitations

Our reported rates were limited by the geography and study period, which occurred prior to the WHO revision to MPN subclassification. Furthermore, this study does not include additional epidemiology measures such as prevalence and survival rate.

## Data Availability

The datasets used and/or analyzed during this study are available from the corresponding author on reasonable request.

## Abbreviations

CML: chronic myeloid leukemia
ET: essential thrombocythemia
JAK2: Janus kinase 2
MPN: myeloproliferative neoplasm
MPN-U: unclassified MPN
PMF: primary myelofibrosis
PV: polycythemia vera
WHO: World Health Organization
